# AmyloDeep: protein language model-based ensemble model for predicting amyloid propensity from the amino acid sequence

**DOI:** 10.1093/bioadv/vbag296

**Published:** 2026-10-06

**Authors:** Alisa Davtyan, Anahit Khachatryan, Rafayel Petrosyan

**Affiliations:** Institute of Physics, Yerevan State University, Yerevan 0025, Armenia; Institute of Physics, Yerevan State University, Yerevan 0025, Armenia; Institute of Physics, Yerevan State University, Yerevan 0025, Armenia; Zaven P. and Sonia Akian College of Science and Engineering, American University of Armenia, Yerevan 0019, Armenia

## Abstract

**Motivation:**

Amyloids are predominantly β-sheet-rich, stable protein structures that can maintain their presence in the human body for multiple years. Amyloid protein aggregates contribute to the development of multiple neurodegenerative diseases, such as Alzheimer’s, Parkinson’s, and Huntington’s, and are involved in different vital functions, such as memory formation and immune system function.

**Results:**

Here, we used advanced machine learning and deep learning techniques to predict amyloid propensity from the amino acid sequence. First, we aggregated labeled amino acid sequence data from multiple sources, obtaining a roughly balanced dataset of 2365 sequences for binary classification. We leveraged that data to both fine-tune the ESM2 model and to train new models based on protein embeddings from ESM2 and UniRep. The predictions from these models were then unified into a single soft voting ensemble model, yielding highly robust and accurate results. We further made a tool where users can provide the amino acid sequence and get the amyloid formation probabilities of different segments of the input sequence.

**Availability and implementation:**

Users can access the light version of AmyloDeep through the web server at https://amylodeep.com/, and the full model is available as a Python package at https://pypi.org/project/amylodeep/.

## 1 Introduction

Amyloids are long filamentous structures typically rich in beta sheets (Chiti and Dobson 2017, Iadanza *et al.* 2018). Toxic amyloids are involved in many neurodegenerative diseases, while functional amyloids are involved in important physiological functions (Otzen and Riek 2019) and can even serve as materials for various applications (Cherny and Gazit 2008, Peña‐Díaz *et al.* 2024). Despite the recent success of protein structure prediction from its amino acid sequence (Jumper *et al.* 2021, Abramson *et al.* 2024, Zheng *et al.* 2026), the prediction of the amyloid-forming regions from the amino acid sequences is still an open question. The fact that the same polypeptide chain can fold into a globular native state and, in other cases, can be part of a long amyloid fibril with vastly different structure is one of the demonstrations of the non-uniqueness of sequence-to-structure correspondence. The identification of the amino acid sequence regions that are prone to amyloid formation is the first important step for their further investigation (Fernandez-Escamilla *et al.* 2004, Conchillo-Solé *et al.* 2007, Goldschmidt *et al.* 2010, Doherty *et al.* 2020). Consequently, the availability of precise and accessible tools for such tasks is of great importance.

Several computational methods have been developed over the years to predict amyloidogenic regions in protein sequences. Physics-based approaches such as TANGO (Fernandez-Escamilla *et al.* 2004) and ZipperDB (Goldschmidt *et al.* 2010) rely on thermodynamic models and steric zipper energetics, respectively, while statistical and machine learning (ML) methods, such as WALTZ (Maurer-Stroh *et al.* 2010), AGGRESCAN (Conchillo-Solé *et al.* 2007), and AMYLPRED2 (Tsolis *et al.* 2013) use experimentally derived propensity scales or consensus predictions from multiple algorithms. More recently, ANuPP (Prabakaran *et al.* 2021) incorporated nucleation kinetics into its predictions. Despite their contributions, these methods share important limitations. The majority of existing tools were developed using relatively small datasets of experimentally validated amyloidogenic peptides, which may not capture the full complexity of amyloid-forming regions. Moreover, most classical methods rely on amino acid propensity scales that were largely calibrated on hydrophobic, β-sheet-prone sequences. As a consequence, they tend to underperform on proteins whose amyloidogenic regions are driven by different physicochemical properties, such as prion-like proteins with low-complexity, polar-rich domains. Also, many existing tools do not use the long-range sequence representations that have become available through recent advances in protein language models (pLMs), which can capture residue-level dependencies beyond what fixed propensity scales encode.

Two important developments in recent years contributed to the creation of our tool. First, thanks to the development of new experimental techniques (Lövestam and Scheres 2022, Garcia-Pardo and Ventura 2024) and efficient protocols, the number of experimentally confirmed amino acid sequences that are shown to form amyloids under *in vivo*-like conditions has grown (Belli *et al.* 2011, Wozniak and Kotulska 2015, Varadi *et al.* 2018, Louros *et al.* 2020, Rawat *et al.* 2020, Sawaya *et al.* 2021, Iglesias *et al.* 2024). It is known that many proteins and peptides can form amyloids under appropriate conditions (Cherny and Gazit 2008, Adler-Abramovich *et al.* 2012). We, however, are mostly interested in amyloidogenesis under physiologically relevant conditions, which is the primary target of most current methodologies (Fernandez-Escamilla *et al.* 2004, Conchillo-Solé *et al.* 2007, Walsh *et al.* 2014, Charoenkwan *et al.* 2022, Planas-Iglesias *et al.* 2024, Gonay *et al.* 2025). Second, the development of novel ML and deep learning algorithms and, more recently, the emergence of pLMs have increasingly transformed the field of protein science (Bordin *et al.* 2023, Weissenow and Rost 2025). pLMs trained exclusively on primary sequence data can learn emergent properties not explicitly provided during training. For instance, these models can infer three-dimensional structural information (Elnaggar *et al.* 2022, Weissenow and Rost 2025) and enable the *de novo* design of proteins with desired functionalities (Madani *et al.* 2023, Hayes *et al.* 2025). This is possible since protein sequences are not random amino acid sequences, and models with transformer-based architectures have proven to be able to learn “protein language grammar” (Bepler and Berger 2021).

Here, we developed a novel, highly accurate pLM-based ensemble model for predicting the amyloid formation probability from the amino acid sequence. We aggregated data from several experimentally verified databases (Belli *et al.* 2011, Wozniak and Kotulska 2015, Varadi *et al.* 2018, Louros *et al.* 2020, Rawat *et al.* 2020, Sawaya *et al.* 2021), used ESM2 (Lin *et al.* 2023) and UniRep (Alley *et al.* 2019) embeddings, and created five binary classification ML models. These were then combined into a single soft voting ensemble model to provide the final probability values. We then developed a tool that provides the aggregation profile for an amino acid sequence with a user-defined running window size.

## 2 Methods

### 2.1 Data aggregation

To obtain a unified dataset for amyloid classification, we integrated data from six experimentally verified databases: CPAD 2.0 (https://web.iitm.ac.in/bioinfo2/cpad2/index.html, Rawat *et al.* 2020), Waltz-DB 2.0 (http://waltzdb.switchlab.org/, Louros *et al.* 2020), Amyloid Atlas (https://people.mbi.ucla.edu/sawaya/amyloidatlas/, Sawaya *et al.* 2021), AmyPro (http://amypro.net; Varadi *et al.* 2018), AmyLoad (http://comprec-lin.iiar.pwr.edu.pl/amyload/, Wozniak and Kotulska 2015), and AmyloBase http://bioserver2.sbsc.unifi.it/AmyloBase.html, Belli *et al.* 2011). These resources varied in file formats and annotation detail, requiring parsing and preprocessing pipelines. When available, UniProt identifiers were used as standard keys to combine entries across datasets. We eliminated repeating sequences, removing entries where identical amyloid-forming regions appeared in multiple databases with matching labels, retaining only one copy. We ended up with 2365 sequences, 1236 labeled as amyloid (these are experimentally validated amyloidogenic peptide segments) and 1129 as non-amyloid.

The dataset was split into three parts: 70% training, 15% validation, and 15% test sets. After performing sequence clustering using MMseqs2 with a 40% sequence identity threshold, we removed the sequences from the test set belonging to clusters that have members in the training set. As a result, the test set was reduced to 305 sequences (these can be found in the Supplementary Information (SI) section Test set, available as supplementary data at *Bioinformatics Advances* online). This test set “Test set 305.csv,” available as supplementary data at *Bioinformatics Advances* online.

### 2.2 Models

Fine-tuned ESM2 150M: for the amyloid classification task, we used the ESM2 pLM (Lin *et al.* 2023). We used a transfer learning approach wherein the pretrained ESM2 parameters remained frozen while only the classification layer was fine-tuned for binary amyloid/non-amyloid classification. The ESM2 model family includes variants of different sizes, ranging from 8 million to 15 billion parameters. For a comprehensive evaluation, we fine-tuned four variants—ESM2-T6-8M, ESM2-T12-35M, ESM2-T30-150M, and ESM2-T33-650M—using the same training protocol and dataset. All models were initialized with pre-trained weights and fine-tuned with a frozen backbone, updating only the classification head parameters. Among the evaluated variants, the ESM2-T30-150M model consistently demonstrated superior performance for our task, achieving the best tradeoff between model capacity and generalization. While the 650M model showed comparable results on training data, it exhibited signs of overfitting and required significantly more computational resources. In contrast, the 150M model provided stable convergence, with higher accuracy and sensitivity. Hence, we selected the 150M model as our final architecture and continued fine-tuning it for additional epochs using early stopping criteria based on validation loss. Hyperparameters such as learning rate, batch size, and dropout rate, as well as the number of epochs, were tuned via grid search within the cross-validation framework. Additionally, we experimented by unfreezing some encoder layers in addition to the classification head of the selected ESM2-T30-150M model. While this setup allowed the model to better capture sequence patterns, it also introduced overfitting. During training, we observed rapid decreases in training loss accompanied by degradation in validation performance, indicating that the model was memorizing the training data rather than generalizing. We tried regularization techniques and hyperparameter tuning, but we were still unable to overcome overfitting. Consequently, only the classification layer was fine-tuned, while the core parameters of the pretrained ESM2-T30-150M model remained unchanged.

Unified representation (UniRep) of sequences combined with a feedforward neural network (FNN): UniRep is a multiplicative LSTM trained on protein sequences to learn a general-purpose, numerical representation (embedding) of proteins directly from sequence data (Alley *et al.* 2019). It generates fixed-length (1900) embeddings for amino acid sequences. We used the UniRep output embeddings and trained an FNN. It consists of five hidden linear layers with ReLU activation functions, and to prevent overfitting, we incorporated dropout layers with a rate of 0.5. This model is the fastest for training and inference and is deployed on our web server as a light version.

ESM2 650M combined with three separate ML models: we have used the pretrained pLM ESM2 650M to extract the embedding layer outputs (the resulting matrix has dimensions of sequence length by 1280) and used them as input features for three separate binary classification models. One of these models is an FNN consisting of five linear layers with ReLU activations, and to prevent overfitting, we incorporated dropout layers with a rate of 0.2. The second classification model is a support vector machine (SVM) classifier implemented with a linear kernel and regularization parameter *C* = 1. The third classification model is an XGBoost classifier configured with 10 estimators, a maximum tree depth of 3, and a learning rate of 0.1.

Each model was validated using five-fold cross-validation to ensure robustness and generalization across different data splits. The cross-validation metrics, as well as training and test metrics, can be seen in SI, available as supplementary data at *Bioinformatics Advances* online. Following the experimentation and tuning phase, the final models were trained from scratch on an A100 GPU for about 30 h.

### 2.3 Soft voting ensemble model

The predictions of the above-described five models were used to construct an ensemble model with soft voting. We evaluated calibration with reliability diagrams and applied either sigmoidal (Platt) or isotonic calibration, selecting the method per model using the reliability plot, expected calibration error, accuracy, and specificity (Niculescu-Mizil and Caruana 2005, Guo *et al.* 2017). As a result, the fine-tuned ESM2 150M and ESM2 650M with SVM were kept as they were, for ESM2 650M with FNN and ESM2 650M with XGBoost, isotonic calibration was applied, and for UniRep with FNN, sigmoidal calibration was applied. The ensemble prediction is the arithmetic mean of the five models’ predicted probabilities.

### 2.4 Feature importance analyses

We conducted SHapley Additive exPlanations (SHAP, Lundberg and Lee 2017) analysis to assess the contribution of each component model to the ensemble predictions and to investigate the extent to which amino acid composition explains the ensemble model’s predictions. First, we applied Kernel SHAP to the soft-voting ensemble, using the predicted probabilities from the five component models as input features, to quantify the relative influence of each model on the final predictions. This demonstrated that all five component models contributed meaningfully to the ensemble prediction. Second, to evaluate the extent to which ensemble predictions can be explained by amino acid composition alone, we constructed a surrogate model trained to approximate the ensemble model’s output using only amino acid frequencies as input features. SHAP analysis was then applied to this surrogate to identify which amino acids most strongly drive predictions and in which direction. The details and the SHAP plots can be found in the SI section SHAP analysis, available as supplementary data at *Bioinformatics Advances* online.

## 3 Results

### 3.1 AmyloDeep ensemble model

The schematic of the AmyloDeep ensemble model is presented in Fig. 1. First, the ESM2 and UniRep models process the input amino acid sequence to generate numerical embeddings. These embeddings then serve as input features for a set of downstream predictor models—FNN, SVM, or XGBoost. After the calibration step, the ensemble model prediction probability is calculated as the arithmetic mean of the probabilities from five binary classification models. The ensemble model’s overall performance is better than that of any of its components.

**Figure 1:**
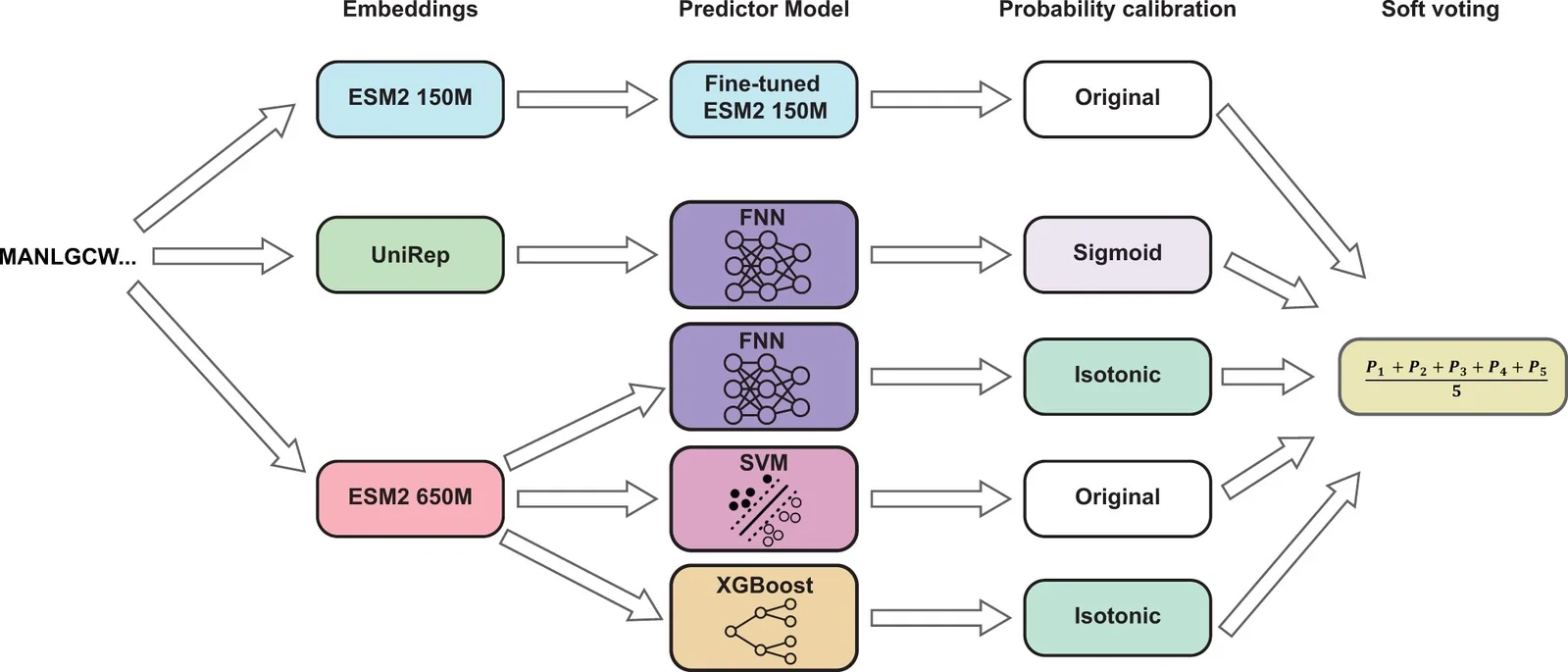
Schematics of AmyloDeep. The input sequence is sent to the ESM2 150M and 650M versions (Lin *et al.* 2023) and to UniRep (Alley *et al.* 2019), from which the embeddings are sent to the predictor models. Afterwards, the appropriate probability calibration is applied, and the arithmetic mean of the resulting probabilities from five models is calculated.

### 3.2 Performance

The main performance metrics for the ensemble model are presented in Table 1. The receiver operating characteristic (ROC) curve and specificity-sensitivity curve obtained on the test set are presented in Fig. 2a and b, respectively. Overall, this shows a very good performance of our classification ensemble model. However, these are the absolute results, and next, we show the comparisons of AmyloDeep with other similar tools.

**Figure 2:**
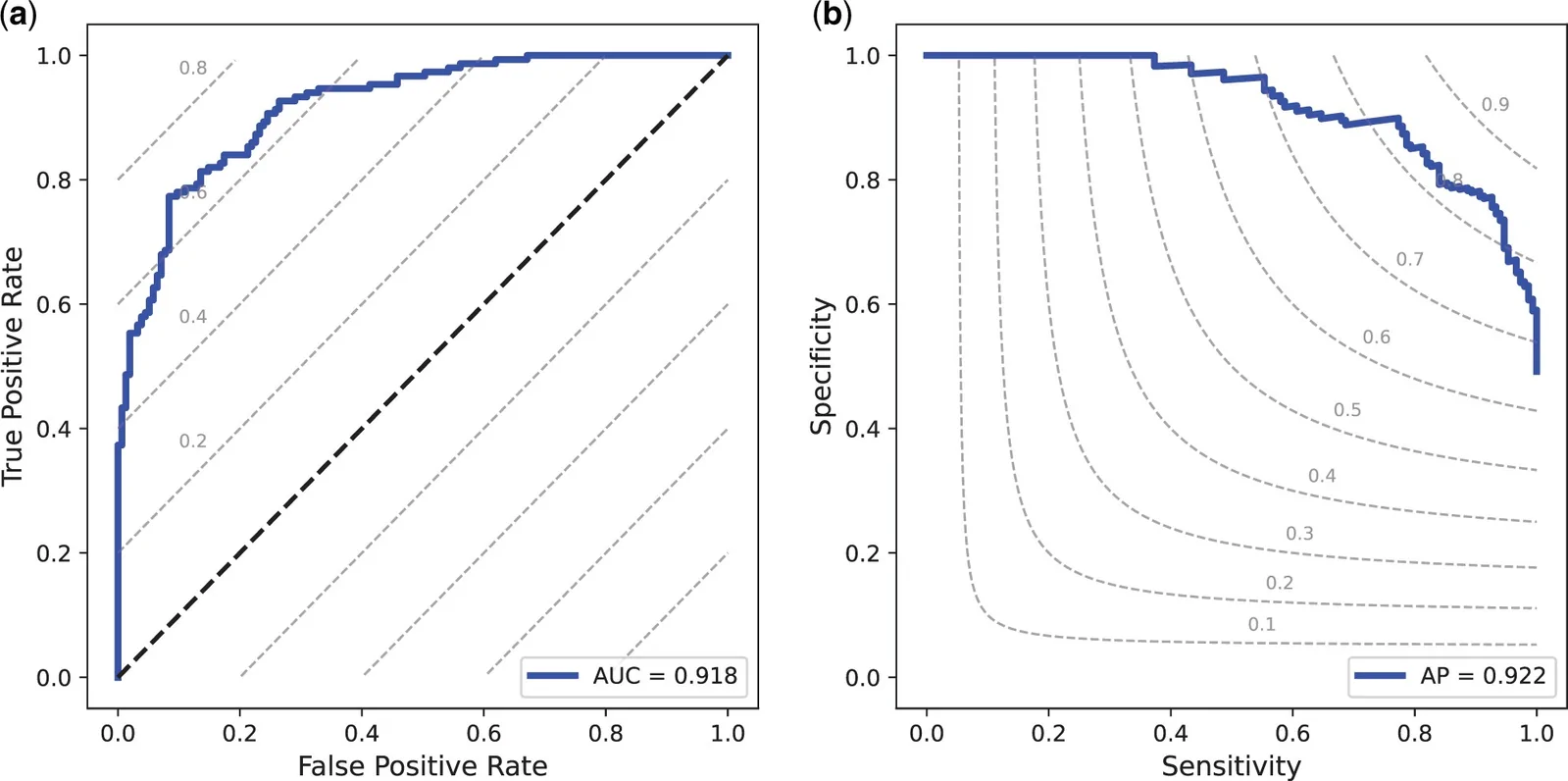
(a) ROC curve obtained on the test set, with an area under the curve being 0.918 TPR·FPR. The dashed lines are iso-performance lines with corresponding TPR–FPR values labeled on them. The black dashed diagonal line corresponds to a random classifier with TPR–FPR = 0. (b) Specificity-sensitivity curve obtained on the test set, the average precision is 0.922. The dashed gray lines are iso-F1 score curves with corresponding F1 scores labeled on them.

**Table 1:** AmyloDeep performance metrics for amyloid class prediction on the test set.

Accuracy (%)	Sensitivity (%)	Specificity (%)	Precision (%)	F1 score (%)	MCC (%)
82.95	82.00	83.87	83.11	82.55	65.89

### 3.3 Comparison with other methods

In this section, we compare the performance of AmyloDeep with several other methods for amyloid propensity prediction. In Fig. 3, we show the precision, sensitivity, F1 score, and accuracy of the Cross-Beta predictor (Gonay *et al.* 2025), AMYPred-FRL (Charoenkwan *et al.* 2022), and AmyloDeep using sequences from our test set that had a length of 15 amino acids or more, since the Cross-Beta predictor provides predictions only on sequences that are at least 15 amino acids long. Thus, we ended up with a test set of 48 sequences. This test set “Reduced test set 48.csv,” available as supplementary data at *Bioinformatics Advances* online. AmyloDeep shows superior performance compared to these models on all of these metrics.

**Figure 3:**
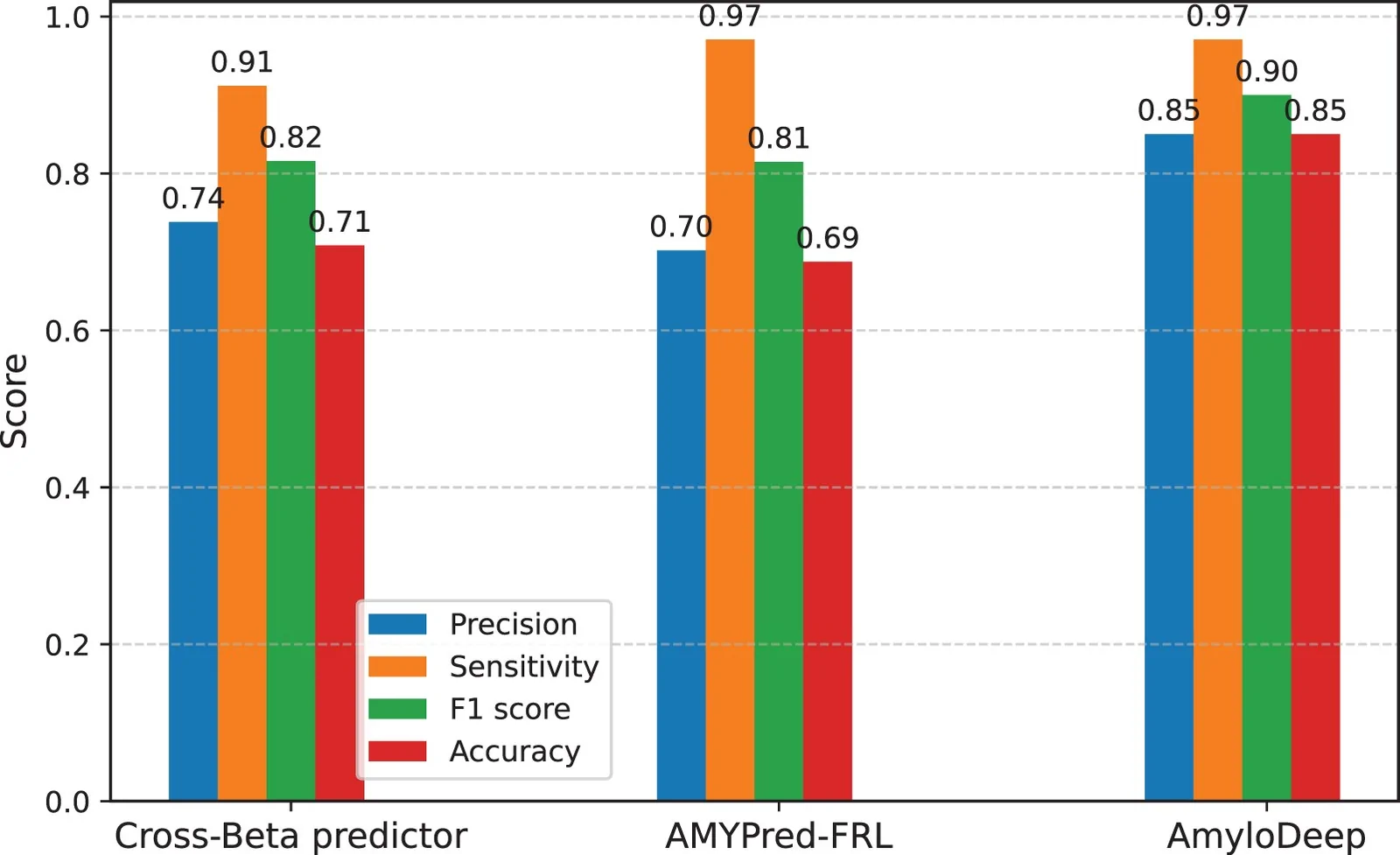
Comparison of AmyloDeep with recently proposed ML-based methods, the cross-beta predictor (Gonay *et al.* 2025) and AMYPred-FRL (Charoenkwan *et al.* 2022). We selected sequences of at least 15 amino acids long from our test set since the cross-beta predictor provides predictions only on sequences that are at least 15 amino acids long.

Based on our ensemble model, we developed a tool that provides an aggregation profile for a given amino acid sequence. Here, users can provide the amino acid sequence, specify the running window size (we tested window sizes ranging from 3 to 15 amino acids and concluded that a window size of 10 was optimal; subsequently, we set it as the default in our tool, but users are free to choose a desired window size from 3 to the full length of the entered sequence) and obtain an aggregation profile. The light version is available through the web server at https://amylodeep.com/, and the full model is available as a Python package https://pypi.org/project/amylodeep/. Next, in Fig. 4, we compare aggregation profiles predicted by AmyloDeep and two widely used tools: ZipperDB (Goldschmidt *et al.* 2010) and AGGRESCAN (Conchillo-Solé *et al.* 2007). For ZipperDB, if Rosetta energy is below -23 kcal/mol, then it predicts higher-than-average aggregation propensity, and if Rosetta energy is below -25 kcal/mol, then we have aggregation-prone regions. Because AmyloDeep returns an amyloid formation probability, we use two decision thresholds: regions with a probability above 0.5 are labeled amyloid-prone, and those with a probability above 0.8 are labeled high-confidence amyloid-prone. In Fig. 4a, we show the aggregation profile for human PrP (UniProt: P04156) with the gray-shaded area corresponding to the amyloid fibril spanning residues 170 − 229 (PDB: 6LNI)․ In this qualitative comparison, we notice that all three tools provide similar profiles and predict the amyloid fibril spanning residues 170 − 229. In Fig. 4b, we show the aggregation profile for human heterogeneous nuclear ribonucleoprotein D-like (UniProt: O14979), with the gray shaded area corresponding to the amyloid fibril spanning residues 345 − 395 (PDB: 7ZIR)․ In contrast to the results shown in Fig. 4a and b demonstrates that AmyloDeep provides a noticeably more accurate prediction of the amyloid fibril spanning residues 345–395 compared to ZipperDB and AGGRESCAN.

**Figure 4:**
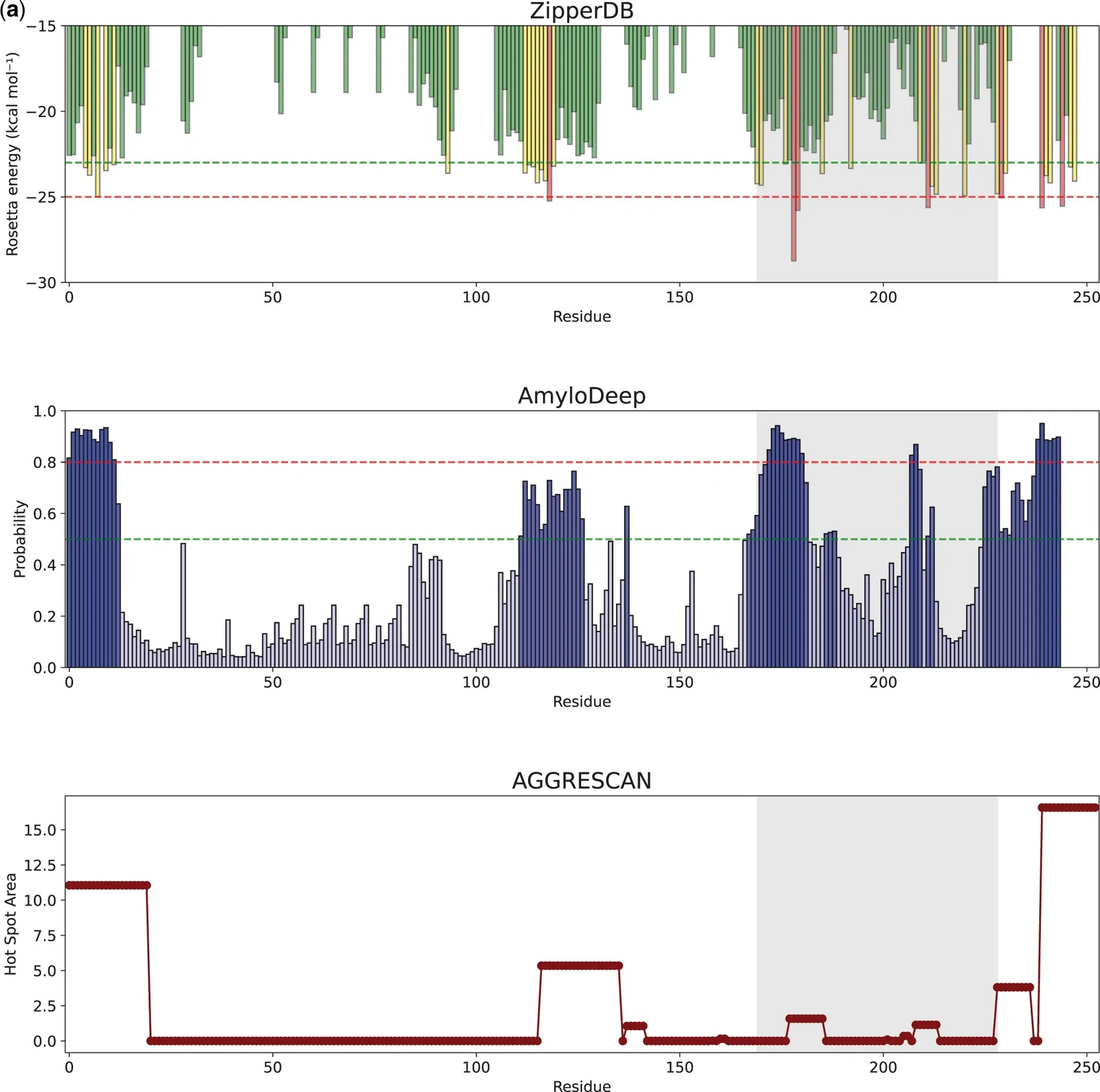

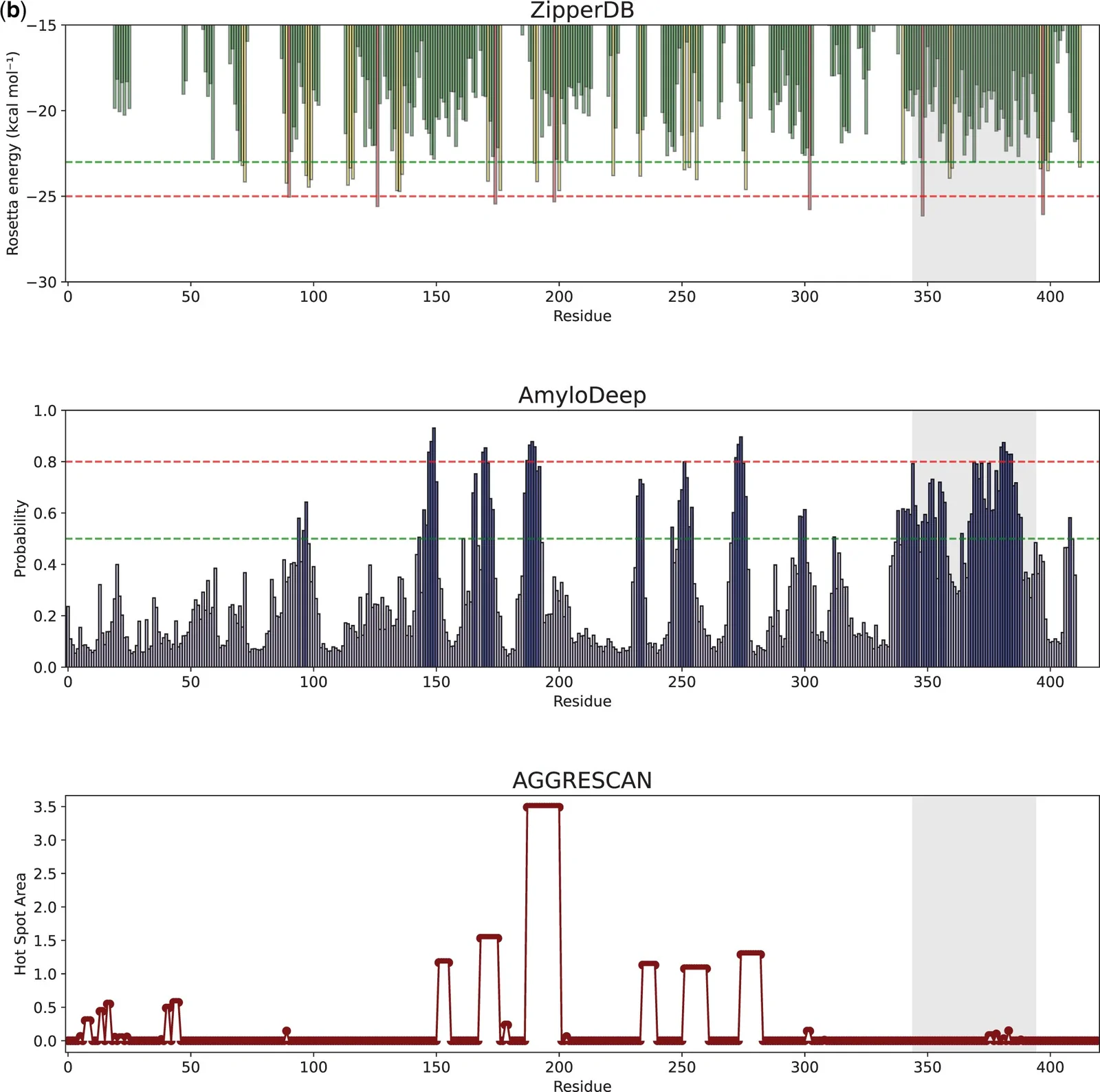
Qualitative comparison of AmyloDeep with well-established methods, ZipperDB (Charoenkwan *et al.* 2022), and AGGRESCAN (Conchillo-Solé *et al.* 2007) using aggregation profiles. (a) We used the human PrP sequence (UniProt: P04156) for comparison. The gray-shaded area corresponds to the amyloid fibril spanning residues 170 − 229 (PDB: 6LNI)․ (b) We used the human heterogeneous nuclear ribonucleoprotein D-like sequence (UniProt: O14979) for comparison. The gray-shaded area corresponds to the amyloid fibril spanning residues 345 − 395 (PDB: 7ZIR). For ZipperDB, the green dashed line shows the threshold value of residues with a high beta-zipper formation propensity, the red dashed line indicates the high-aggregation propensity threshold, the yellow bars indicate residues with a higher-than-average aggregation propensity, and the red bars indicate aggregation-prone regions. For AmyloDeep with 10 amino acids running window, the green and red dashed lines indicate the amyloid formation probability thresholds of 0.5 and 0.8, respectively, and the corresponding bars are colored with darker shades of blue.

## 4 Discussion

We first briefly describe how the methods compared in this study were developed. AGGRESCAN uses the aggregation propensity values per amino acid derived from experiments using single-point mutations at the central position of Aβ42 (De Groot *et al.* 2006, Conchillo-Solé *et al.* 2007). Then it uses a running window of size 5, 7, 9, or 11, depending on the input sequence length (Conchillo-Solé *et al.* 2007). The latest version, AGGRESCAN4D (Bárcenas *et al.* 2024), predicts pH-dependent aggregation and has the protein 3D structure as an input. ZipperDB uses Rosetta energy calculated based on hexapeptide zipper crystal structures; hence, ZipperDB has a running window size of six amino acids for aggregation profiles (Goldschmidt *et al.* 2010). The updated version of ZipperDB is based on a neural network that generates scores for individual hexapeptide sequences (https://zipperdb.mbi.ucla.edu/info). Cross-beta predictor is an Extra Trees classifier trained on the Cross-Beta DB, a database based on cross-beta structures (Gonay *et al.* 2025). It has a minimum running window size of 15 amino acids for the aggregation profile. AMYPred-FRL is a stacked ensemble model trained on the Amy dataset (Charoenkwan *et al.* 2022), where only sequences with 50 amino acids or more are present, and AMYPred-FRL currently does not provide an aggregation profile option. In contrast to ZipperDB, which targets the steric zipper motif, a specific structural feature not shared by all amyloids, or the cross-beta predictor, which focuses on cross-β forming regions, our method is not based on the underlying fibril architecture. By training on all available experimentally labeled sequence data, we believe our tool captures a broader range of amyloidogenic sequence determinants.

Our experiments with AlphaFold 2 and 3 (Jumper *et al.* 2021, Abramson *et al.* 2024) showed that they performed poorly in predicting amyloid structures. There could be several reasons for this. First, although in recent years many amyloid fibril structures have been determined with CryoEM and other methods and deposited into the PDB, we still have few such structures [we have around 100 000 unique protein structures (https://www.rcsb.org/statistics/growth/unique-protein-sequences) and only around 800 amyloid fibril structures (https://people.mbi.ucla.edu/sawaya/amyloidatlas/) as of September 2026], resulting in limited training data. A single amino acid sequence may yield either a native-like globular structure or an amyloid fibril; hence, the same sequence can produce at least two drastically different structures. AmyloDeep and similar models could become a part of protein structure prediction tools, where the model could get amyloid formation probability from AmyloDeep and use that information, e.g. to provide two structures to the users for a given sequence: one globular native folded structure, and the other, an amyloid fibril structure.

We used three different embeddings (ESM2 150M, ESM2 650M, and UniRep; Alley *et al.* 2019, Lin *et al.* 2023) and five different predictive models. This gives an advantage in the sense that different encoders can grasp different features and properties of an amino acid sequence, which could be important for predicting amyloid formation. Using diverse model families (FNN, SVM, XGBoost, fine-tuned ESM2 150M) provides diversity in a similar sense as for the encoders mentioned previously. Finally, the soft voting integrates their probabilities, producing a stable, consensus decision. The number and size of the models and encoders, on the other hand, are limited by available computational resources for training and inference. Inference time is a key user-facing metric. Among our five models, the fastest in training and inference is UniRep combined with FNN. This is because the UniRep embeddings are smaller. Independent of the length of the amino acid sequence, it returns a 1900-dimensional vector, while the embeddings of ESM2 depend on the length of the amino acid sequence (it is one of the dimensions of the embedding matrix). Hence, we deploy UniRep combined with FNN as a light version on our web server.

The ESM3 model and its embeddings recently became publicly accessible, allowing for custom fine-tuning (Hayes *et al.* 2025). We evaluated the feasibility of fine-tuning the open-access variant ESM3-sm-open-v1, which contains approximately 1.4 billion parameters. However, we found that ESM3-sm-open-v1 underperformed compared to ESM2 150M for our specific use case, so we concluded that ESM2 was the more effective choice for our use case.

That said, we recognize several limitations of our method. First, the limited amount of data used for training, validation, and testing, and the relatively large fraction of short sequences, may constrain the model’s ability to capture the full diversity of amyloidogenic sequence features. This is a common bottleneck in the amyloid prediction field, as experimentally validated amyloidogenic proteins and regions remain relatively scarce compared to other areas of protein science. Second, full-model inference can be quite computationally intensive and time-consuming, particularly when processing large-scale datasets. This arises primarily from the embedding generation step, where each input sequence must be passed through the pLM. Furthermore, this step scales linearly with sequence length when the sliding window approach is used, in which the pLM must generate embeddings for every overlapping fragment across the entire protein sequence.

In summary, we developed a novel model for amyloid propensity prediction from the amino acid sequence that showed state-of-the-art performance and made it accessible through a web server and a Python package. Similar tools have been used for determining fibril-forming segments that nucleate aggregation for further experimental studies (Brumshtein *et al.* 2018, Doherty *et al.* 2020) and for the development of a database for proteome aggregation predictions (Badaczewska-Dawid *et al.* 2024). Further development could be the consideration of the external conditions, such as pH, on amyloid propensity. Such steps have already been taken (Bárcenas *et al.* 2024). These steps can also be useful for protein structure prediction tools, as the structure of a protein also depends on external conditions.

## Supplementary Material

vbag296_Supplementary_Data

## Data Availability

The test data underlying this article are available as supplementary data at *Bioinformatics Advances* online. Full data can be made available upon reasonable request.
