# Supplementary material for: AmyloDeep: protein language model-based ensemble model for predicting amyloid propensity from the amino acid sequence
Source: Bioinform Adv. 2026 Oct 6;6(1):vbag296. doi: 10.1093/bioadv/vbag296 (PMC13730270; doi:10.1093/bioadv/vbag296)
Supplement: Supplementary file 1 [file vbag296_supplementary_data.zip › SI Prediction of amyloid propensity 7 no yellow proofs.pdf]

Supplementary Information

AmyloDeep: pLM-based ensemble model for predicting amyloid propensity from the amino acid sequence

Alisa Davtyan[1], Anahit Khachatryan[1], Rafayel Petrosyan[1, 2, *]

[1] *Institute of Physics, Yerevan State University, 0025 Yerevan, Armenia*
[2] *Zaven P. and Sonia Akian College of Science and Engineering, American University of Armenia, 0019 Yerevan, Armenia*

Below, we provide the performance metrics and cross-validation metrics for the five individual models that are part of the ensemble model. For each model, 5-fold cross-validation was used, and the final performance metrics presented below are for amyloid class prediction averaged across all validation folds.

Fine-tuned ESM2 150M

	Accuracy	Sensitivity	Specificity	Precision	F1 score	MCC
Test	82.95 %	84.00 %	81.94 %	81.82 %	82.89 %	65.93 %
Validation	85.63 %	86.44 %	84.83 %	85.00 %	85.71 %	71.28 %
Train	83.43 %	85.91 %	80.75 %	82.83 %	84.34 %	66.82 %

Cross-validation Fine-tuned ESM2 150M

	Fold 1	Fold 2	Fold 3	Fold 4	Fold 5	Mean±SD
Accuracy	80.97 %	80.66 %	83.08 %	79.76 %	81.21 %	81.14±1.09 %
Sensitivity	86.05 %	82.56 %	82.56 %	80.23 %	84.80 %	83.24±2.01 %
Precision	79.14 %	80.68 %	84.52 %	80.70 %	80.11 %	81.03±1.84 %
F1 score	82.45 %	81.61 %	83.53 %	80.47 %	82.39 %	82.09±1.02 %

As mentioned in the methods section for ESM2-T30-150M, only the classification layer was fine-tuned, while the core parameters of the pretrained model remained unchanged. We have previously partially fine-tuned ESM2-T30-150M by unfreezing the last two encoder layers and the classification head. In that case model overfitted: training loss dropped quickly while validation performance declined, indicating memorization rather than generalization.

[*] Correspondence to: rafayel.petrosyan@aua.am

UniRep combined with FNN

	Accuracy	Sensitivity	Specificity	Precision	F1 score	MCC
Test	81.64 %	80.67 %	82.58 %	81.76 %	81.21 %	63.27 %
Validation	84.79 %	83.05 %	86.52 %	85.96 %	84.48 %	69.61 %
Train	87.55 %	87.43 %	87.67 %	88.46 %	87.94 %	75.07 %

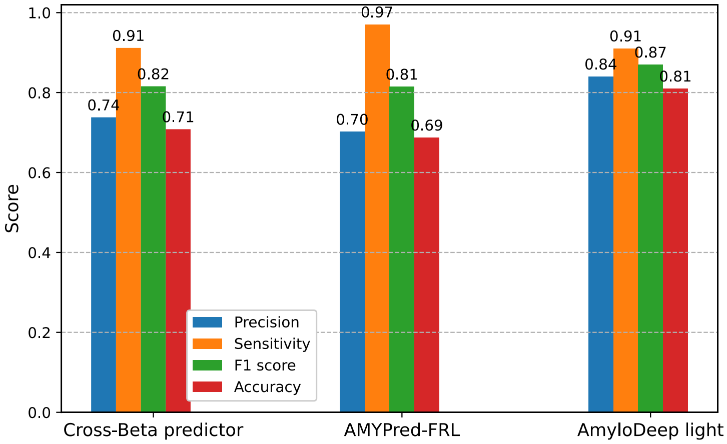

The figure on the left shows the comparison of UniRep combined with the FNN model, which is our light model with the Cross-Besta predictor and AMYPred-FRL on 48 test sequences that are not shorter than 15 residues.

Cross-validation UniRep combined with FNN

	Fold 1	Fold 2	Fold 3	Fold 4	Fold 5	Mean±SD
Accuracy	82.78 %	80.66 %	80.66 %	80.66 %	81.52 %	81.26±0.83 %
Sensitivity	84.88 %	82.56 %	79.65 %	79.07 %	82.46 %	81.72±2.12 %
Precision	82.49 %	80.68 %	82.53 %	82.93 %	81.98 %	82.12±0.78 %
F1 score	83.67 %	81.61 %	81.07 %	80.95 %	82.22 %	81.90±0.99 %

ESM2 650M combined with FNN

	Accuracy	Sensitivity	Specificity	Precision	F1 score	MCC
Test	80.66 %	79.33 %	81.94 %	80.95 %	80.13 %	61.30 %
Validation	85.07 %	86.44 %	83.71 %	84.07 %	85.24 %	70.17 %
Train	98.37 %	97.67 %	99.12 %	99.17 %	98.42 %	96.74 %

Cross-validation ESM2 650M combined with FNN

	Fold 1	Fold 2	Fold 3	Fold 4	Fold 5	Mean±SD
Accuracy	80.97 %	80.66 %	80.66 %	81.27 %	82.12 %	81.14±0.54 %
Sensitivity	86.63 %	75.00 %	74.42 %	76.74 %	79.53 %	78.46±4.45 %
Precision	78.84 %	86.00 %	86.49 %	85.71 %	85.00 %	84.41±2.83 %
F1 score	82.55 %	80.12 %	80.00 %	80.98 %	82.18 %	81.17±1.04 %

ESM2 650M combined with SVM

	Accuracy	Sensitivity	Specificity	Precision	F1 score	MCC
Test	81.64 %	80.00 %	83.23 %	82.19 %	81.08 %	63.27 %
Validation	84.51 %	84.18 %	84.83 %	84.66 %	84.42 %	69.01 %
Train	85.91 %	87.19 %	84.53 %	85.89 %	86.54 %	71.78 %

Cross-validation ESM2 650M combined with SVM

	Fold 1	Fold 2	Fold 3	Fold 4	Fold 5	Mean±SD
Accuracy	80.66 %	80.97 %	83.08 %	84.29 %	82.42 %	82.28±1.34 %
Sensitivity	86.05 %	82.56 %	81.40 %	85.47 %	83.04 %	83.70±1.77 %
Precision	78.72 %	81.14 %	85.37 %	84.48 %	83.04 %	82.55±2.39 %
F1 score	82.22 %	81.84 %	83.33 %	84.97 %	83.04 %	83.08±1.09 %

ESM2 650M combined with XGBoost

	Accuracy	Sensitivity	Specificity	Precision	F1 score	MCC
Test	80.98 %	78.67 %	83.23 %	81.94 %	80.27 %	61.98 %
Validation	83.94 %	85.31 %	82.58 %	82.97 %	84.12 %	67.92 %
Train	84.82 %	88.71 %	80.63 %	83.19 %	85.86 %	69.69 %

Cross-validation ESM2 650M combined with XGBoost

	Fold 1	Fold 2	Fold 3	Fold 4	Fold 5	Mean±SD
Accuracy	79.76 %	83.99 %	83.99 %	81.57 %	80.61 %	81.98±1.75 %
Sensitivity	83.72 %	84.88 %	83.72 %	81.98 %	82.46 %	83.35±1.06 %
Precision	78.69 %	84.39 %	85.21 %	82.46 %	80.57 %	82.26±2.43 %
F1 score	81.13 %	84.64 %	84.46 %	82.22 %	81.50 %	82.79±1.50 %

Aggregation profile comparisons of several proteins from three different categories

Intrinsically disordered proteins

Tau UniProt: P10636 PDB: 5O3L

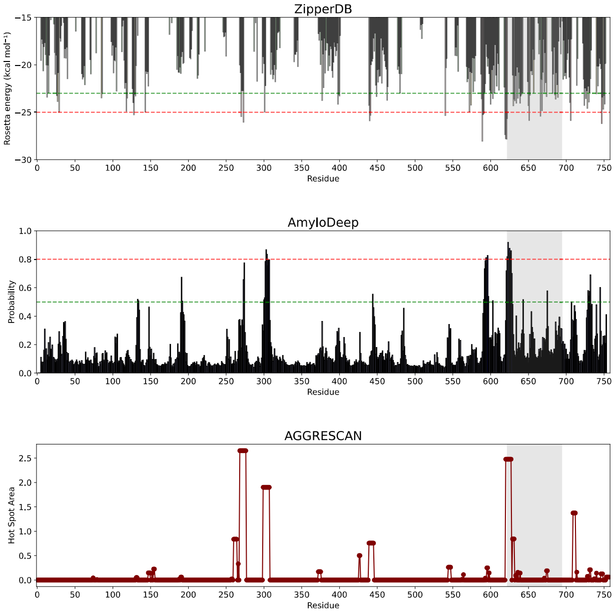

Amyloid-β UniProt: P05067 PDB: 5OQV

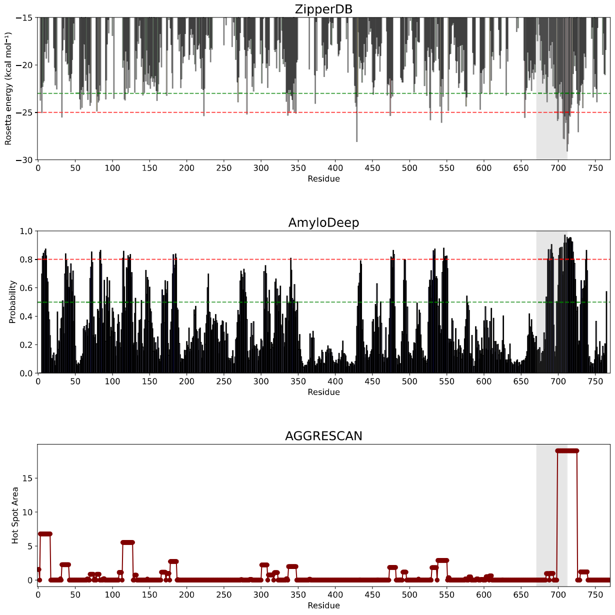

Amylin/IAPP UniProt: P10997 PDB: 6VW2

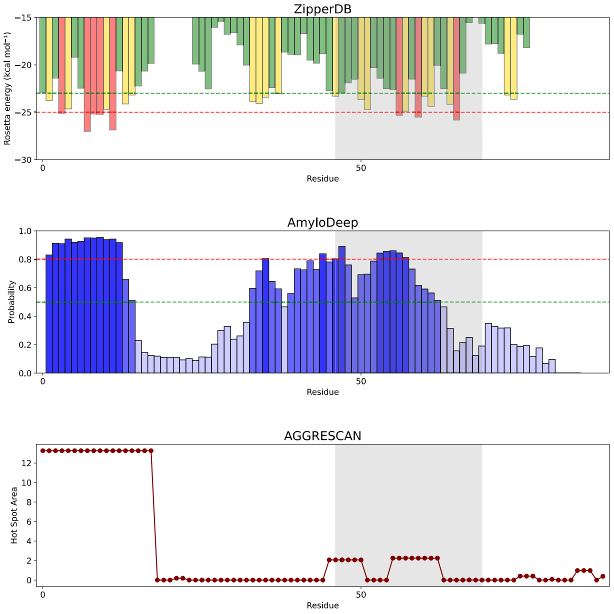

For the intrinsically disordered proteins (IDPs) shown above, all three methods identify amyloidogenic regions to a certain degree and show mostly consistent predictions, which is expected since these proteins contain hydrophobic, β-sheet-prone aggregation segments that classical propensity scales were designed to detect.

Globular proteins

TRR UniProt: P02766 PDB: 6SDZ

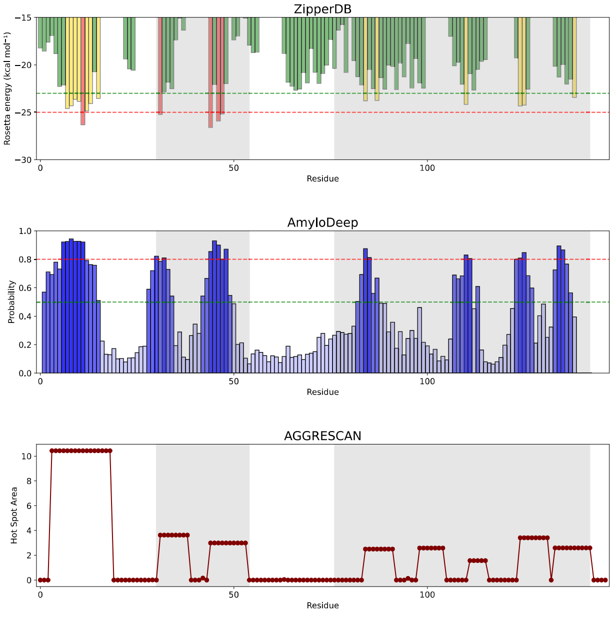

β2-microglobulin UniProt: P61769 PDB: 6GK3

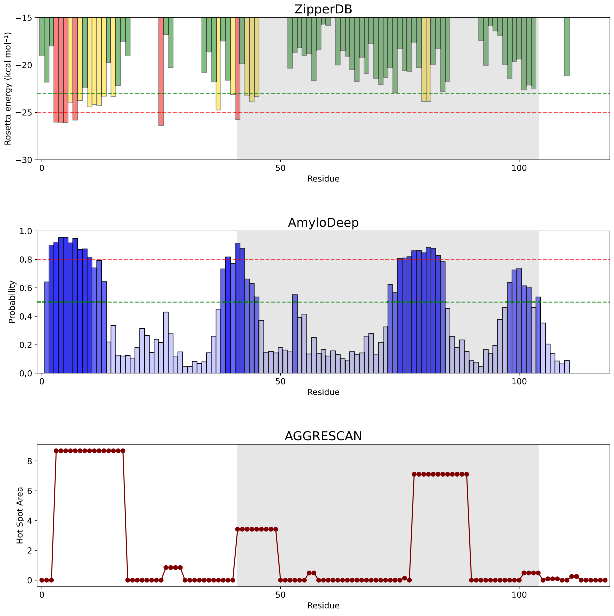

Lysozyme C UniProt: P61626 PDB: 8R4A

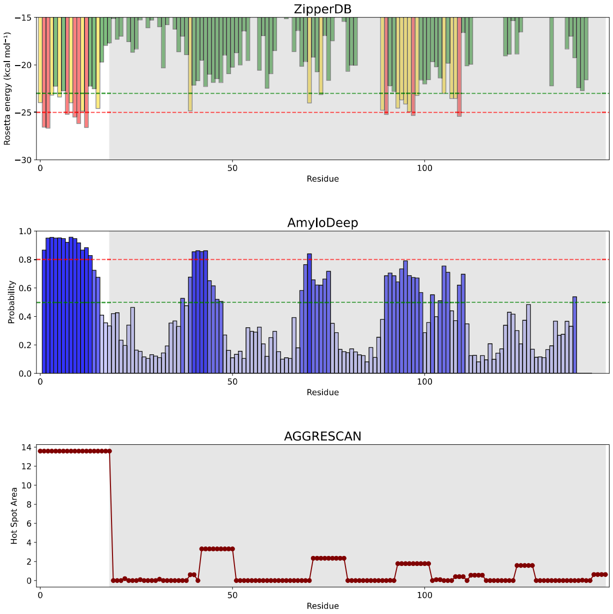

SAA1 UniProt: P0DJI8 PDB: 6MS

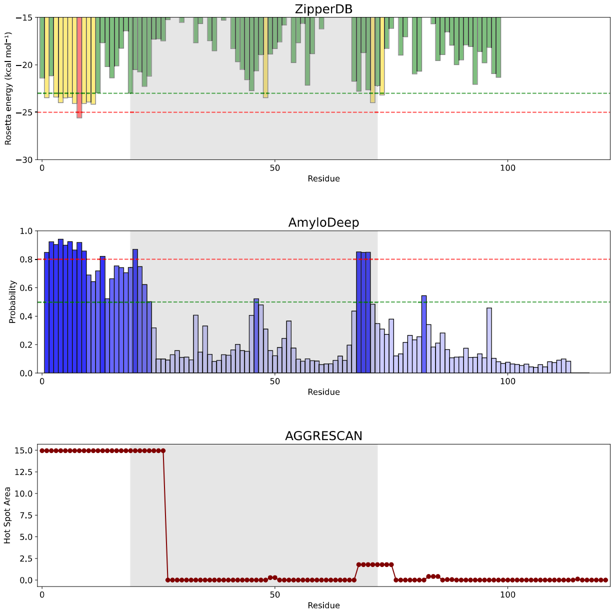

For the globular protein examples shown above, differences between the methods are noticeable. Particularly, AmyloDeep does a better job of predicting the fibril core region (shaded) in TTR and β2-microglobulin than ZipperDB, and for SAA1, all methods struggle.

Prion-like proteins

TDP-43 UniProt: Q13148 PDB: 7PY2

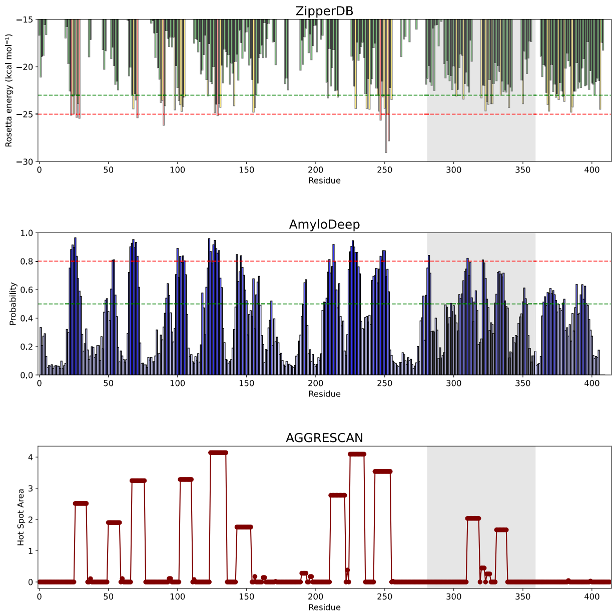

FUS UniProt: P35637 PDB: 5W3N

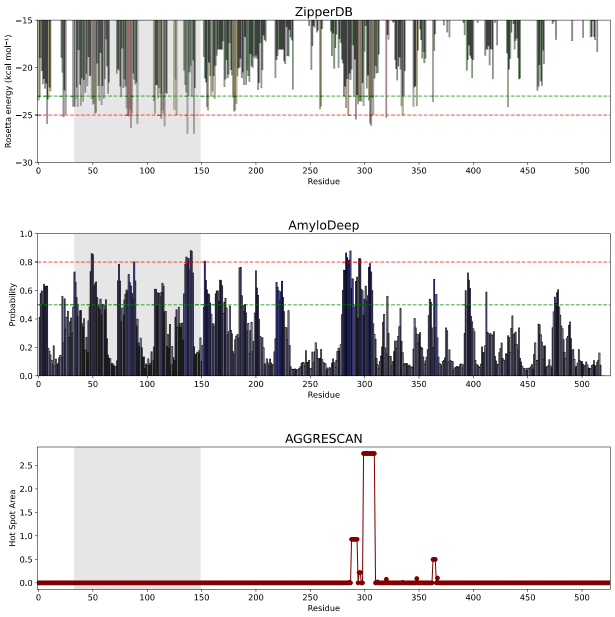

For the prion-like proteins, the difference between methods is evident. The amyloid cores of TDP-43 and FUS lie within their low-complexity domains, where hydrophobic and aromatic residues are rare, on which classical predictors rely. AGGRESCAN does not detect the fibril core region in FUS and shows relatively weak hot spots in TDP-43.

SHapley Additive exPlanations (SHAP) analysis

We performed SHAP analyses in two ways: first, we investigated the influence of each model on the final ensemble predictions. We applied Kernel SHAP to the soft-voting ensemble model's output and used the probabilities produced by the five models as input features. The figure below on the left shows the mean absolute SHAP values, from which we can see the relative influence of each model on final ensemble predictions. The figure below on the right shows the beeswarm plot for each model. Each dot in this plot represents one sequence from the test set. Dots to the right of zero pushed the prediction toward amyloidogenic, while dots to the

left pushed the prediction toward non-amyloidogenic. The further from zero, the stronger the influence.

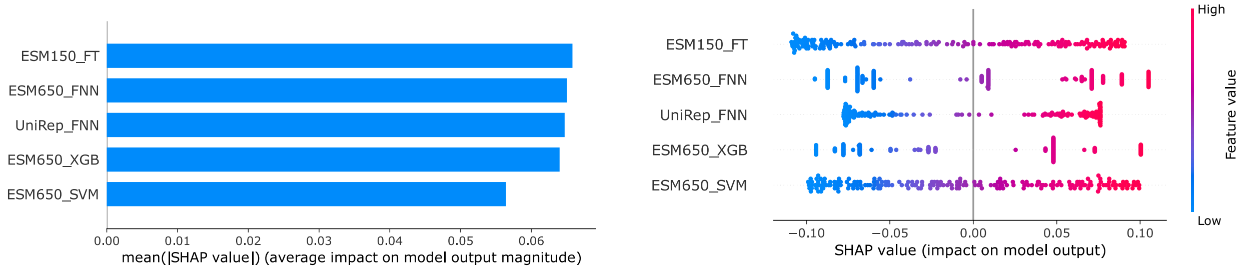

Next, we did a SHAP analysis using amino acid frequencies as features. For this, we created a matrix of frequencies for each amino acid for each sequence in the test set. For example, for the AVVGGGGK sequence of length 8, we have freq_A = 1/8, freq_V = 2/8, freq_G = 4/8, and freq_K = 1/8, and all other amino acid frequencies are 0. As in our case, all the model inputs are encoded sequences, the ensemble model never directly sees the frequencies of the amino acid, to overcome this we create a XGBRegressor-based surrogate model, where the input is the matrix of the frequencies we created above, and the output is the ensemble model's prediction probabilities (after soft voting), after training it we get $R^2 = 0.9781$, which means that using only amino acid frequencies the surrogate can explain approximately 97.4 % of the variation in ensemble model predictions. Nevertheless, the advantage of pLM-based representations becomes apparent in the aggregation profile analysis on full-length proteins, where AmyloDeep correctly identifies amyloidogenic regions, for example, in prion-like proteins that are missed by composition-based tools.

The figure below on the left shows the mean absolute SHAP values, from which we can see the relative influence of each amino acid frequency on the surrogate model predictions. The figure below on the right shows the beeswarm plot for each amino acid frequency. Each dot in

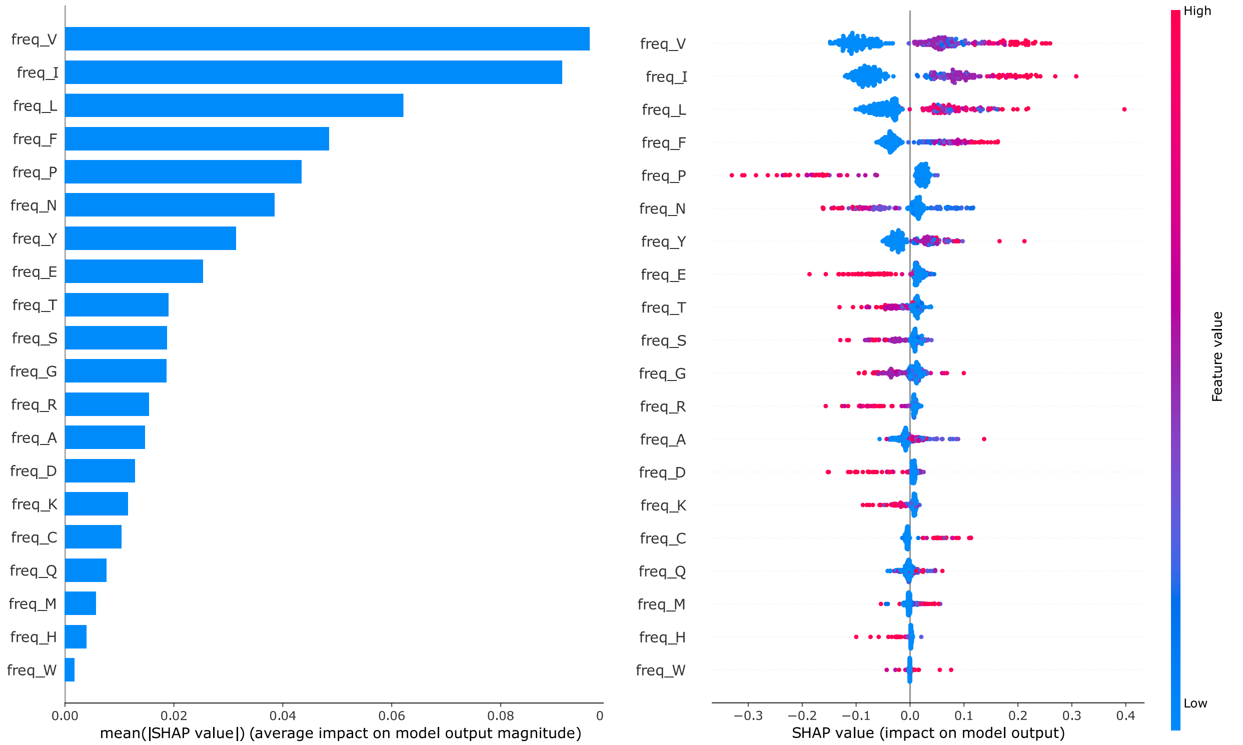

this plot represents one sequence from the test set. Dots to the right of zero pushed the prediction toward amyloidogenic, while dots to the left pushed the prediction toward non-amyloidogenic. The further from zero, the stronger the influence. For example, for freq_V, red dots cluster on the right, blue dots on the left. This means sequences with high valine content are pushed toward amyloidogenic predictions, and sequences with low valine content are pushed toward non-amyloidogenic predictions. For freq_P, the pattern is reversed: high proline content pushes toward non-amyloidogenic predictions.
